# Ferroptosis contributes to diabetes-induced visual pathway neuronal damage via iron accumulation and GPX4 inactivation

**DOI:** 10.1007/s11011-024-01398-5

**Published:** 2024-07-30

**Authors:** Bowen Wang, Ying Jin, Xuan Ouyang, Ru Zhu, Xinghua Wang, Shuang Li, Fagang Jiang

**Affiliations:** 1 Department of Ophthalmology, Union Hospital, Tongji Medical College, Huazhong University of Science and Technology, Wuhan, 430022 China; 2 Department of Ophthalmology, the Central Hospital of Wuhan, Tongji Medical College, Huazhong University of Science and Technology, Wuhan, 430014 China; 3 Aier Eye Hospital of Wuhan University, Wuhan, 430060 China

**Keywords:** Diabetes, Visual pathway, Ferroptosis, GPX4, Diabetes retinopathy

## Abstract

The damage of the diabetic visual pathway is one of the main causes of blindness in diabetic patients. Visual pathways include anatomic parts from the retina to the occipital lobe. This study investigated the involvement of ferroptosis, a planned cell death brought on by the buildup of free iron in cells, in the impairment of visual pathways in diabetes mellitus. Streptozotocin (STZ) was used to construct a diabetic rat model. Pathological and ultrastructural changes of the occipital lobe, retina, and optic nerve were observed by Hematoxylin–Eosin (HE) staining and transmission electron microscopy (TEM). The expressions of Neuronal nuclei (NeuN), Glial fibrillary acidic protein (GFAP), and Glutathione Peroxidase 4 (GPX4) in the occipital lobe and retina were detected by immunofluorescence, and Western Blotting was used to identify the NeuN GFAP and GPX4 expressions in the occipital lobe. Iron content in the occipital lobe and retina was detected by Iron Assay Kit. The success rate of the diabetic rat model was 93.3%. In the diabetic group, the cells of the occipital lobe and retina were arranged disorderly, and the boundaries were unclear. The membrane of the occipital lobe, retina, and optic nerve was broken, some vacuoles were observed, mitochondrial morphology was changed, swelling was observed, and the mitochondrial ridge disappeared. There was a large increase in GFAP expression and iron concentration and a significant decrease in the expression of NeuN, and GPX4 in the retina and occipital lobe. Ferroptosis plays an important role in visual pathway damage in diabetes, and GPX4 regulates this process.

**Supplementary Information:**

The online version contains supplementary material available at 10.1007/s11011-024-01398-5.

## Introduction

Diabetes is one of the fastest-growing diseases worldwide, projected to affect 693 million adults by 2045 (Cole and Florez 2020). The epidemic of diabetes mellitus and its complications poses a major global health threat (Zheng et al. 2018). In recent years, the research on diabetic optic neuropathy and central nervous system complications has been increasing (Palavicini et al. 2020; Feldman et al. 2019), and the influence of diabetes on visual pathways has become a research focus. The visual pathway is an important anatomical and physiological basis for vision formation, including all the functional structures that transmit visual signals from retinal ganglion cells optic nerve to the occipital visual center (Ungewiss et al. 2020). Previous studies have confirmed that there are lesions of the optic nerve and visual pathway in the early stage of diabetes (Sachdeva 2021). Animal experiments showed that there were significant morphological changes in all functional structures of visual pathways in diabetic rats. All of them imply that early-stage diabetes damages the structure and function of visual circuits.

Ferroptosis is a novel procedural death mechanism that has been described recently. It differs significantly from the conventional death mode in terms of cell morphology and function, primarily exhibiting mitochondrial shrinkage and elevated lipid peroxidation (Jiang et al. 2021). In age-related macular degeneration (Zhao et al. 2021) and retinitis pigmentosa (Yang et al. 2021), iron accumulation and decreased Glutathione Peroxidase 4/ Glutathione (GPX4/GSH) levels lead to iron death in retinitis pigmentosa and photoreceptor cells. In recent years, more and more studies have shown that ferroptosis plays an important regulatory role in the occurrence and development of diabetes-related complications, including diabetic nephropathy, osteoporosis, myocardial ischemia, and reperfusion (He et al. 2022; Yang and Yang 2022). However, it has not been reported whether ferroptosis is involved in the neuronal death of the visual pathway induced by diabetes. Considering that hyperglycemia and abnormal energy metabolism are involved in the multi-system complications associated with diabetes, we believe that ferroptosis is also involved in diabetes-induced neuronal death in the visual pathway.

Based on these, the present study constructs a diabetic rat model in Sprague Dawley male rats to investigate the role of ferroptosis in diabetic optic neuropathy.

## Methods and materials

### Animals

All experimental procedures were approved by Wuhan Servicebio Technology Co., Ltd., China (application id: Servicebio Animal Welfare No.2023008). A total of 30 specific pathogen-free (SPF) male SD rats (8–10 w old) with a body mass of 200–250 g were purchased from Wuhan Servicebio Technology Co., Ltd. (Wuhan, China). The rats were kept in the SPF laboratory of the experimental animal center at the Tongji Medical College, Huazhong University of Science and Technology, and allowed free access to food and water under 22–25℃ temperature and 50–70% humidity conditions with a 12 h light/dark cycle.

### Construction of the diabetic rat model

**Fig. 1:**
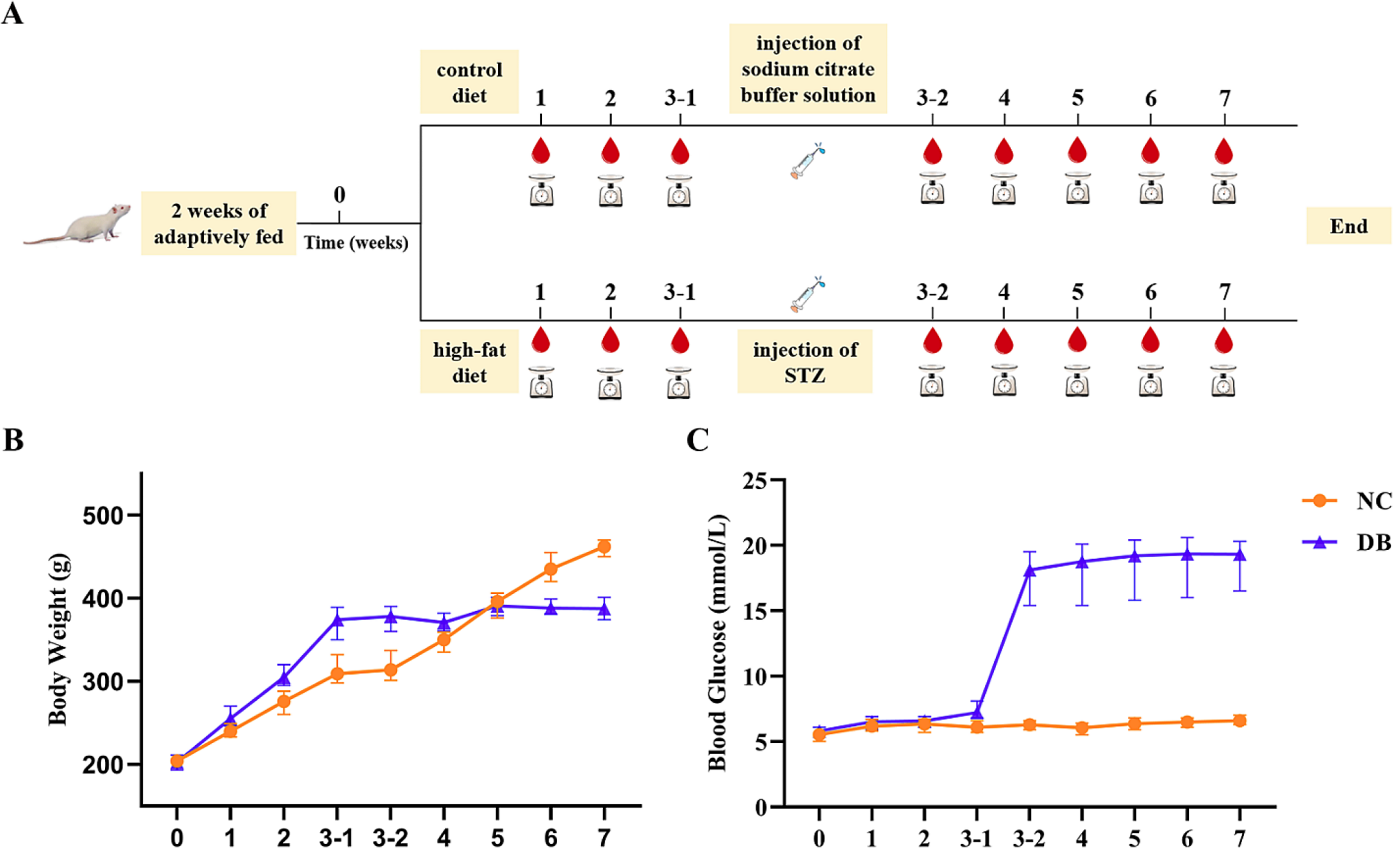
Timeline and related parameters of rats during diabetic modeling. (**A**) Timeline of diabetic rat modeling during the 7 w. Body weight () and non-fasting blood glucose concentration () were tested each w. (**B**) Average body weight during the 7 w. (**B**) Non-fasting blood glucose concentration across the 7-w time course. Data are represented as the mean and range for n = 15 in the negative control (NC) group and n = 15 in the diabetic (DB) group. 3–1: The first time for measurement in w 3 (before injection). 3–2: The second time for measurement in w 3 (after injection). NC: Negative control, DB: Diabetes

The rats were adaptively fed for 2 w. All rats were randomly divided into a normal group and a diabetic group, including 15 rats in the normal group and 15 rats in the diabetic group. The rats in the normal group were maintained on a control diet, while the diabetic group received a high-fat diet (MD12033, Moubaili, Wuhan, China) containing 60% kcal as fat. Body weight and non-fasting blood glucose concentration were detected once per w. The blood glucose collected from the rat tail veins was measured using a standard glucometer (OneTouch UltraVue, Shanghai, China). 3 w later, the rats in the diabetic group received one intraperitoneal injection of streptozotocin (STZ) (40 mg/kg) dissolved in sodium citrate buffer solution, while the normal rats were intraperitoneally injected with the same volume of sodium citrate buffer solution after undergoing 12 h of fasting. Body weight and non-fasting blood glucose concentration were measured each w for the next 4 w (Fig. 1A). The rats with a blood glucose concentration higher than 16.7 mM were selected for subsequent operations.

### Hematoxylin–Eosin (HE) staining

The retina and occipital lobes were fixed in 4% paraformaldehyde for 24 h, dehydrated, and embedded in paraffin. 8 μm thick sections were cut with a vibratome (HistoCore MULTICUT, Leica, USA). The sections were deparaffinized sequentially in xylene (Sinopharm Chemical Reagent Co., Ltd., China), absolute ethanol (Sinopharm Chemical Reagent Co., Ltd, China), 90% alcohol, and 75% alcohol, after which they were washed with tap water. Staining the sections with hematoxylin (H9627-25G, Sigma-Aldrich, USA) and eosin (D12621, Xiya reagent, Linyi, China). The sections were dehydrated in absolute ethanol and xylene until transparent and sealed with neutral gum (G8590, Solarbio, Beijing, China).

### Immunofluorescent staining

Cerebral occipital lobes and retinas were fixed and sectioned to a thickness of 8 μm (paraffin section for immunofluorescence staining) through the disc with a vibratome (HistoCore MULTICUT, Leica, USA). The sections were permeabilized with 0.3% Triton X-100 in phosphate buffer saline (PBS) for 1 h, washed three times in PBS, blocked in buffer (5% goat serum, 2% BSA, and 0.1% Tween-20 in PBS) for 1 h, and incubated overnight with primary antibodies against NeuN (1:200, 26,975–1-AP, Proteintech, Wuhan, China), GFAP (1:200, 16,825–1-AP, Proteintech, Wuhan, China) and GPX4 (1:500, 67,763–1-Ig, Proteintech, Wuhan, China) antibodies at 4 °C overnight, followed by species-specific secondary fluorescent antibodies. After washing by PBS three times, we applied coverslips to the sections with DAPI (G1012, Servicebio, Wuhan, China) and anti-fluorescence quencher (G1401, Servicebio, Wuhan, China) before imaging with a laser confocal scanning microscopy (IX51, OLYMPUS, Japan).

### Transmission Electron Microscopy (TEM)

Freshly enucleated eyeballs and brain tissue were immediately put into precooled 2.5% glutaraldehyde (Sinopharm Chemical Reagent Co., Ltd, China) for 12 h at 4℃. The retinas, optic nerves, and cerebral occipital lobes were carefully and completely separated in a new glutaraldehyde solution for 24 h at 4 °C. Then, the fixed retinas, optic nerves, and cerebral occipital lobes were processed for TEM imaging following a standard protocol. The mitochondria were viewed under a transmission electron microscope (Tecnai G2 20 TWIN, FEI, American), and the mitochondria with ferroptosis features were recorded.

### Western Botting (WB)

Radioimmunoprecipitation assay lysis buffer was added, the supernatant was collected, and the protein concentration was detected using a bicinchoninic acid kit. Proteins were separated by sodium dodecyl sulfate–polyacrylamide gel electrophoresis, transferred to polyvinylidene fluoride membranes, blocked with 5% bovine serum albumin for 1 h at room temperature, and incubated with primary antibody overnight at 4 °C. The primary antibody used were NeuN (1:500, 26,975–1-AP, proteintech, China), GFAP (1:500, 16,825–1-AP, proteintech, China), GPX4 (1:500, T56959, Abmart, China) and GAPDH (1:10,000, A19056, Abclonal, China). After incubation with horseradish peroxidase-labeled goat anti-rabbit secondary antibody (1:3000, GB23303, Servicebio, China) for 1 h at 37 °C, the protein intensity was detected using electrochemiluminescence chemiluminescence reagent and analyzed using ImageJ software.

### Iron Parameters

Cerebral occipital lobe and retinal iron levels were measured using an Iron Assay Kit (HY1212G, HYCEZMBIO, China) according to the manufacturer's instructions. After the reaction solution was configured, the absorbance value at 562 nm was read by a multifunctional enzyme reader.

### Statistical Analysis

GraphPad Prism 9.0 and SPSS27.0 software were used for data analysis, and the control group and each experimental group were compared, respectively. A *t*-test was applied to analyze the difference between the two groups of data. Shapiro–Wilk Test is used to determine whether the distribution is normal, and Levene's Test is used to conduct a homogeneity analysis of variance. If the distribution is normal and the variance is homogeneous, an independent sample T-test can be conducted. Mann–Whitney rank sum test was used for non-compliance. The results were expressed as mean ± standard deviation. When the *P*-value was < 0.05, the difference was considered significant.

## Results

### Parameters of the diabetic model

The initial weight of the rats in each group was 200–250 g. After 3 w, the rats on the high-fat diet were 16.8% heavier compared with the rats on normal chow (370 *vs*. 316 g). Compared to control rats, diabetic groups had a decreased body weight after the injection of STZ at w 7 (*P* < 0.0001), respectively (Fig. 1B). Non-fasting blood glucose concentrations were assessed once per w for the 7-w duration. After the injection of STZ, the non-fasting blood glucose of rats was significantly improved (*P* < 0.0001) (Fig. 1C). 14 of the diabetic group rats had non-fasting blood glucose higher than 16.7 mM, suggesting the modeling success rate was 93.3%.

### Pathological changes are observed in the retinal and occipital lobes

**Fig. 2:**
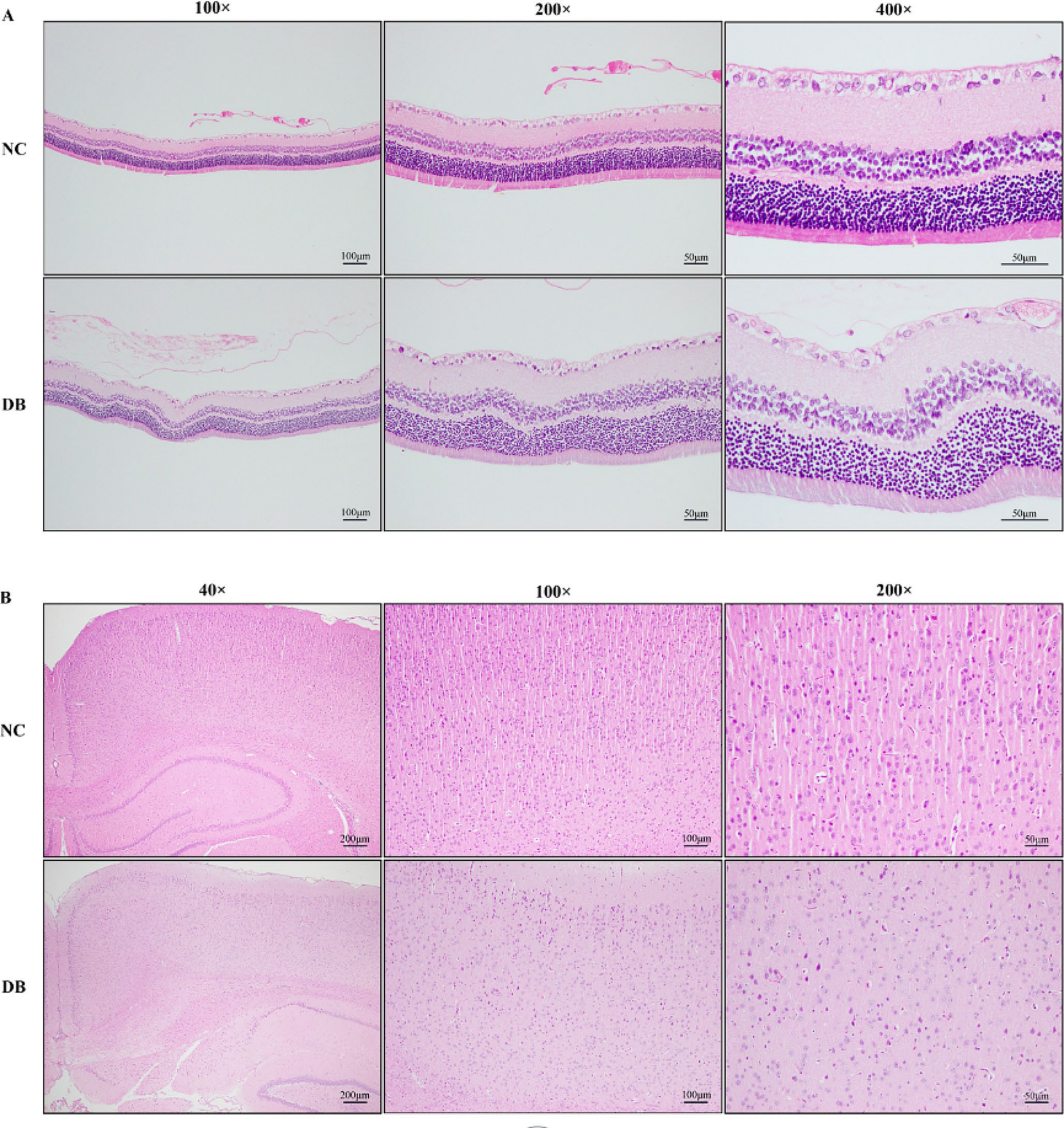
Optical microscopy images of rat retinas and occipital lobes (the HE staining). (**A**) HE staining of retinas in the control group and diabetic group. (**B**) HE staining of occipital lobes in the control group and diabetic group. HE: Hematoxylin–Eosin. NC: Negative control, DB: Diabetes

After 4 w of injection, the retinas and bilateral occipital lobes of rats were obtained, and HE staining was performed. As shown in Fig. 2A, in the control group, the retinal tissue of each layer was arranged neatly, and the retinal ganglion layer, inner nuclear layer (INL), and outer nuclear layer (ONL) were clearly visible. Retinal ganglion cells are the innermost monolayer of cells with large nuclei and light staining. The morphology of the INL and ONL, which were composed of multi-layered cells with small round nuclei and dark nuclear staining, were similar. In the diabetic group, the retina was mildly edematous, with vacuolar degeneration and loose stroma in the retinal ganglion cell layer and INL. The pathological changes of occipital lobes were observed in Fig. 2B. In the control group, the occipital lobe tissue cells were intact, the cells were arranged neatly, and the outline was clear. In the diabetic group, the nuclei were atrophic and aggregated, the cells were arranged disorderly, and the cell contour was unclear.

### Ultrastructure observation of retinal, optic nerve, and occipital lobe

**Fig. 3:**
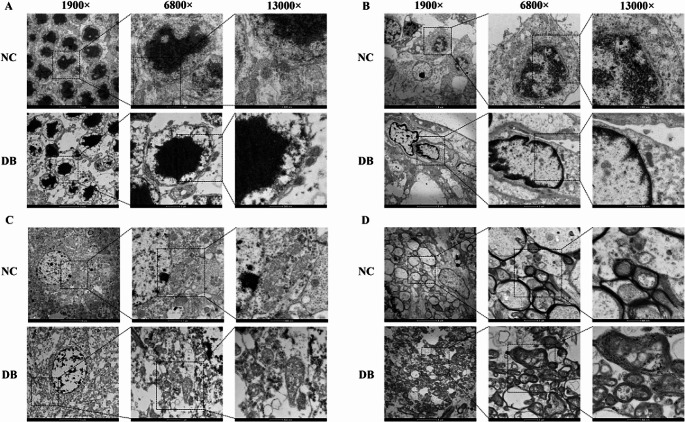
Ultrastructure of the occipital lobe, retina, and optic nerve in control and diabetic group. (**A**-**B**) TEM images of retinal photoreceptor cells and retinal ganglion cells in the control and diabetic group. (**C**) TEM images of occipital neurons in the control and diabetic groups. (**D**) TEM images of optic neurons in the control and diabetic groups. TEM: Transmission electron microscopy. NC: Negative control, DB: Diabetes

After 4 w of injection, the retinas, optic nerves, and bilateral occipital lobes of rats were obtained. The results of TEM are shown in Fig. 3, which represents the ultrastructure of retinal photoreceptor cells (Fig. 3A), retinal ganglion cells (Fig. 3B), occipital neurons (Fig. 3C), and optic neurons (Fig. 3D). In the control group, the cell membrane was intact without rupture, the mitochondrial morphology was normal, the mitochondrial cristae were clear, and no obvious shrinkage, swelling, or vacuoles were observed. In the diabetic group, the cell membrane broke and vesiculated, mitochondria atrophied, mitochondrial ridge decreased or disappeared, membrane density increased, and nucleus morphology was normal, but chromatin agglutination was lacking. Meanwhile, the mitochondria became smaller, and the membrane density increased.

### Relationship among ferroptosis and retinal neurons under diabetic conditions

**Fig. 4:**
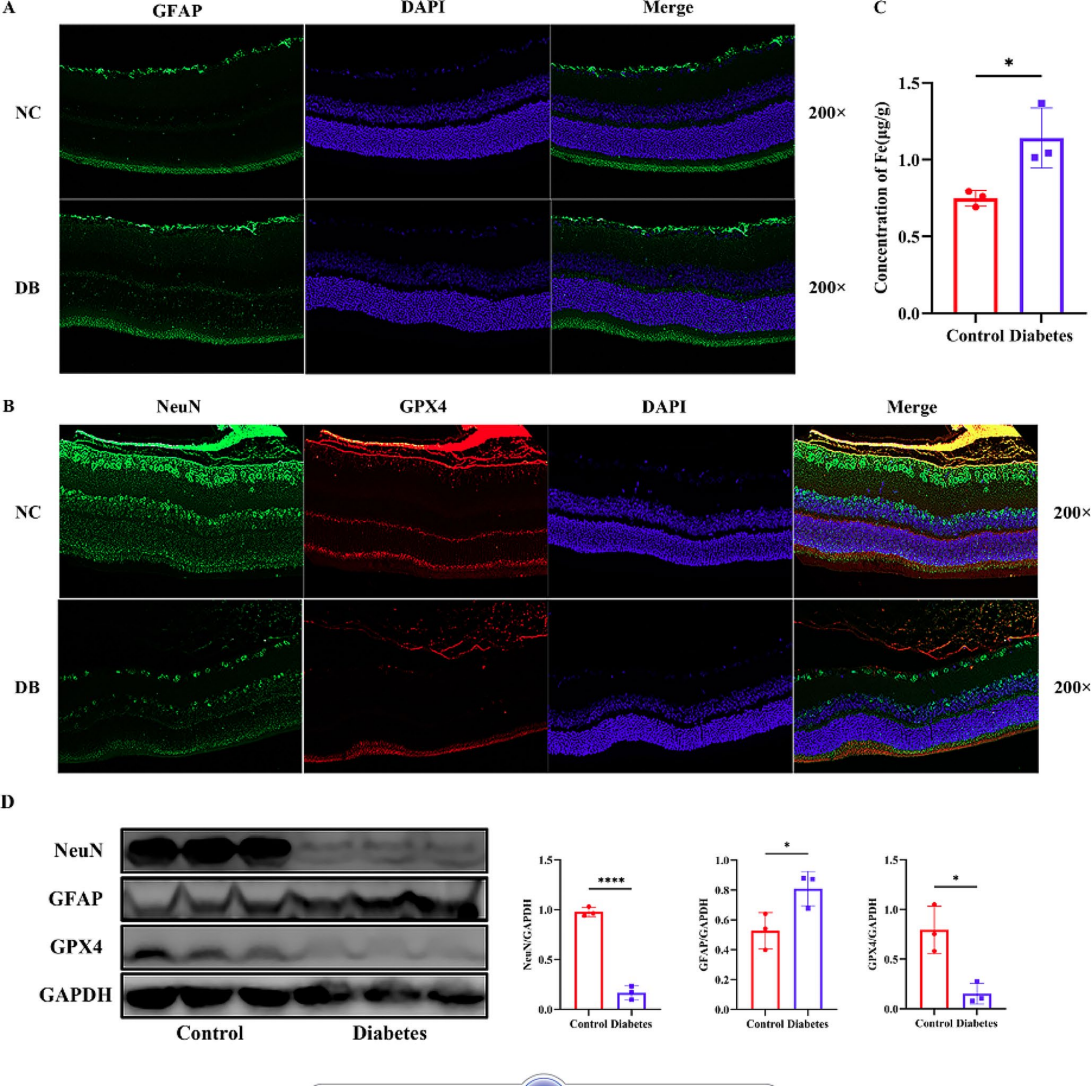
The expression of GFAP, NeuN, GPX4, and iron content testing in the retina in the control and diabetic groups. (**A**) Immunofluorescence for GFAP expression in the retina in the control and diabetic group. (**B**) Immunofluorescence for NeuN and GPX4 expression in the retina in the control and diabetic group. (**C**) Iron content in the retina in the control and diabetic group. (**D**) WB results for GFAP, NeuN, and GPX4 in the control and diabetic group. NC: Negative control, DB: Diabetes, GFAP: Glial fibrillary acidic protein, NeuN: Neuronal nuclei, GPX4: Glutathione peroxidase 4. Data are presented as mean ± SEM (n = 3). *P < 0.05, ****P < 0.0001

After 4 w of injection, the retinas of rats were obtained. Immunofluorescence assay and Western Blotting were used to test the expression of NeuN, GFAP\ and GPX4 in the control and diabetic group. As shown in Fig. 4, NeuN and GFAP appeared green fluorescently; the expression of NeuN was significantly decreased, and the expression of GFAP was increased. NeuN showed a decrease in the inner plexiform layer (IPL), INL, outer plexiform layer (OPL), and ONL. GFAP increased especially in INL, ONL and inner/outer segment (IS/OS). GPX4 showed red fluorescence, which was significantly decreased in OPL and IS/OS. The results of Western Blotting presented the same trend.

### Relationship among ferroptosis and occipital neurons under diabetic conditions

**Fig. 5:**
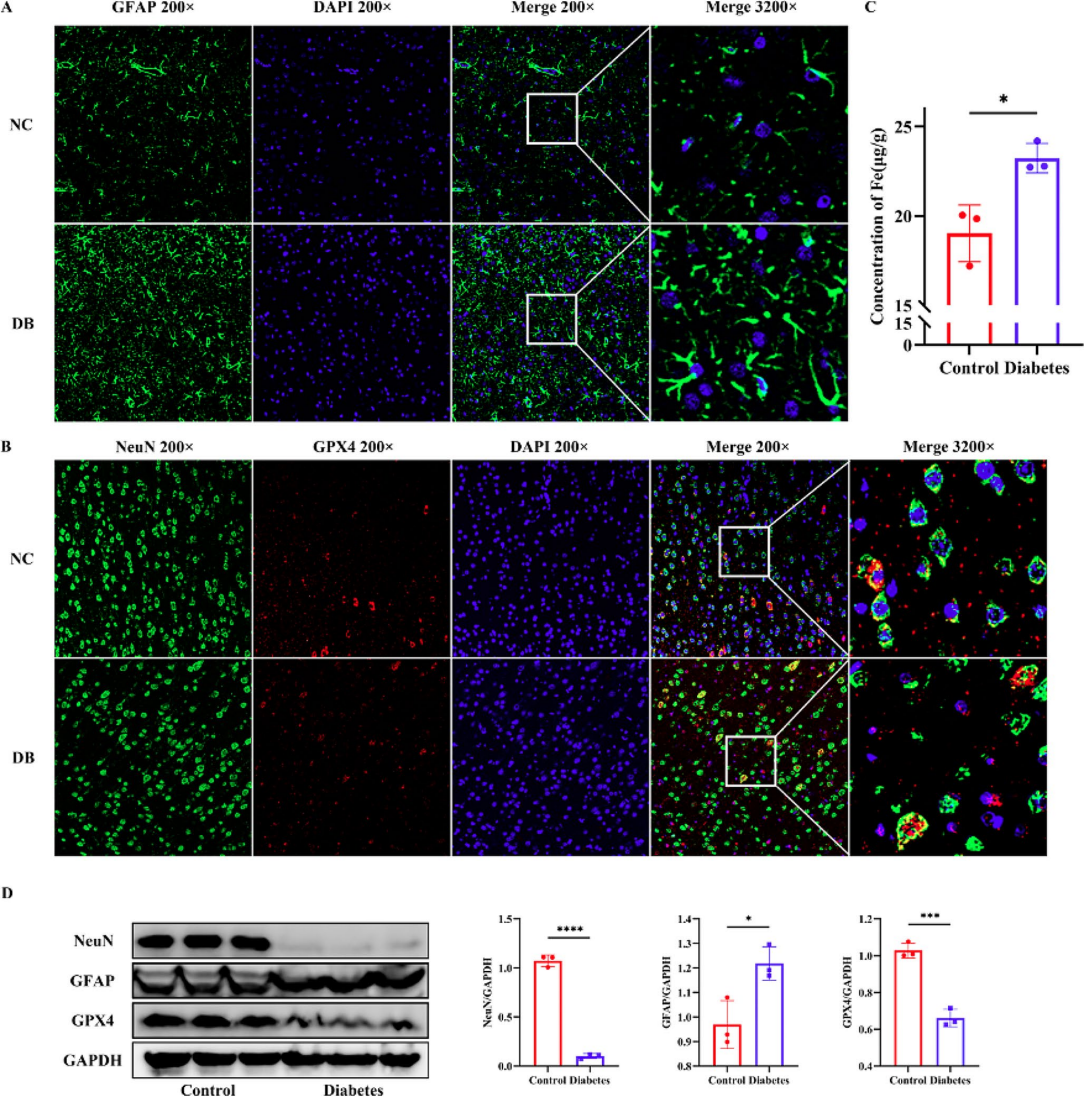
Expression of GFAP, NeuN, GPX4, and iron content testing in the occipital lobe in the control and diabetic group. (**A**) Immunofluorescence for GFAP expression in the occipital lobe in the control and diabetic group. (**B**) Immunofluorescence for NeuN and GPX4 expression in the occipital lobe in control and diabetic group. (**C**) Iron content in the occipital lobe in the control and diabetic groups. (**D**) WB results for GFAP, NeuN, and GPX4 in the control and diabetic groups. NC: Negative control, DB: Diabetes, GFAP: Glial fibrillary acidic protein, NeuN: Neuronal nuclei, GPX4: Glutathione Peroxidase 4, WB: Western Blotting. Data are presented as mean ± SEM (n = 3). *P < 0.05, ***P < 0.001, ****P < 0.0001

After 4 w of injection, bilateral occipital lobes of rats were obtained. Immunofluorescence assay and Western Blotting were used to test the expression of NeuN, GFAP, and GPX4 in the control and diabetic group. As shown in Fig. 5, NeuN and GFAP appeared green fluorescence, and the expression of NeuN was significantly decreased, while the expression of GFAP was significantly increased. GPX4 showed red fluorescence, which also significantly decreased. Protein expression of NeuN and GPX4 showed attenuation in the diabetic group, and GFAP expression in the diabetic group fairly increased.

### Iron levels measurement of retinas and occipital lobes

Cerebral occipital lobes and retinal iron levels were measured after 4 w of injection. As shown in Figs. 4C and 5C, the iron levels of retinas and occipital lobes in the diabetic group were distinctly decreased.

## Discussion

The prevalence of diabetes is increasing rapidly worldwide, with type 2 diabetes accounting for 90% of all cases worldwide among people with diabetes (Kowluru 2020). Diabetic retinopathy is a major microvascular complication of diabetes and one of the leading causes of acquired blindness in adults worldwide (Li et al. 2018). Also, pathophysiological changes in different parts of the visual pathway are closely related to visual impairment in diabetic patients (Ho et al. 2015). The most common complication of diabetes is neuropathy, including distal symmetric polyneuropathy and diabetic optic neuropathy. Thinning of the retinal ganglion cell layer and retinal nerve fiber layer was detected in early diabetic patients (Teng et al. 2018), and functional changes of retinal photoreceptor cells, bipolar cells, and anoplectic cells were already present before microangiopathy was found in diabetic animal models. Studies have shown that early loss of retinal ganglion cells and occipital visual center neurons in diabetic rats is accompanied by an imbalance of metabolic substances in the retina and occipital cortex. Our study examined the expression of NeuN and GFAP in the retina and occipital lobe, the starting and ending points of the visual pathway, and found that the expression level of NeuN in the diabetic group was significantly reduced, while GFAP showed an opposite trend. NeuN is a recognized "marker" for detecting post-mitotic neurons that produce neuron-specific antibodies. Immunostaining evidence indicates that NeuN is distributed in the nuclei of mature neurons in almost all parts of the vertebrate nervous system (Duan et al. 2016). GFAP is a major intermediate filament protein in mature astrocytes and an important component of the cytoskeleton during astrocyte development (Middeldorp and Hol 2011). Our results suggested that there was neuronal damage and astrocyte hyperplasia in the visual pathway in the diabetic group. However, the specific mechanism of the loss and dysfunction of neurons in the visual pathway caused by diabetes has not been elucidated, and the death of functional neurons is the landmark event of the abnormal function of the visual pathway.

Neuronal death is an inevitable and important link in the pathophysiological process of nervous system injury caused by diabetes, which marks the end of neuronal life. The traditional death modes include apoptosis, necrosis, autophagy, and pyroptosis (Tang et al. 2021). Ferroptosis, proposed by Dixon in 2012 (Dixon et al. 2012), is a non-apoptotic form of cell death characterized by iron-dependent lipid peroxidation and metabolic constraints (Seibt et al. 2019), which can be inhibited by iron chelating agents. Suo et al. found that ferroptosis may be related to the death of N-methyl-d-aspartate-induced neuronal degeneration in the retina (Suo et al. 2022). In terms of mechanism, lipid peroxidation, GSH depletion, and ferrous iron accumulation are the main markers of ferroptotic cell death progression (Totsuka et al. 2019). Currently, ferroptosis has been demonstrated to be inextricably linked to several types of neurological disorders (Qin et al. 2022). Cells dying by ferroptosis exhibit a distinct bioenergetic signature, including oncosis (despite unaffected nuclei) and aberrant mitochondrial morphology accompanied by outer membrane rupture as well as cellular disintegration, therefore lacking typical histomorphological hallmarks of apoptosis and other forms of regulated cell death (Seibt et al. 2019). Through TEM, we found that there was cell membrane breakage, vacuolation, mitochondrial shrinkage, and morphological changes in all parts of the visual pathway (including the retina, optic nerve, and occipital lobe) in the diabetic group, which were consistent with the changes of ferroptosis, indicating that ferroptosis existed in the visual pathway in the case of diabetes.

GPX4 is the key regulator of ferroptosis. The mainstay of ferroptosis is the generation of specific phospholipid hydroperoxides in the presence of catalytically active iron, which is endogenously counteracted by the system xc − /GSH/GPX4 axis (Dixon et al. 2012; Yang et al. 2014; Friedmann Angeli et al. 2014). Disordered iron metabolism (especially increased ferrous iron) is the initiating factor of ferroptosis (Yao et al. 2023). Free ferrous, a solid oxidation factor, produces hydroxyl radicals through the Fenton reaction. These unstable hydroxyl radicals can oxidize lipid metabolites, such as polyunsaturated fatty acids, into cytotoxic lipid peroxides (Jenkins et al. 2020; Masaldan et al. 2019). Ferroptosis occurs when lethal lipid peroxides exceed the upper limit of cellular clearance, in which the GPX4/GSH axis plays an important role (Stockwell et al. 2017; Yan et al. 2020). Thus, we detected the iron level and the expression of GPX4 to verify the presence of ferroptosis in the retina and occipital lobe further. In the diabetic group, iron content was significantly increased, indicating that free ferrous accumulated in the retina and occipital lobe, thus inducing the process of ferroptosis. Meanwhile, GPX4 expression was significantly decreased, indicating excessive consumption of GPX4.

In the present study, there were some certain limitations in the experimental design and implementation. Firstly, we only observed the retina, optic nerve and occipital visual cortex, but we did not track and observe other functional structures of the visual pathway, such as superior colliculus (SC) and the lateral geniculate nucleus (LGN). For the overall concept of visual pathway, each functional structure should be evaluated besides the beginning and end of the visual pathway. Secondly, we did not perform the functional test to detect the visual pathways abnormalities induced by diabetes, due to the lack of visual electrophysiological devices for small animals. However, we had to focused on the morphological observations on the optic nerve and occipital visual cortex concerning ferroptosis. Thirdly, the specific mechanism by which ferroptosis regulates visual pathway damage in diabetes, such as GPX4/xCT/SLC7A11 and NAD(P)H/FSP1/CoQ10 systems, needs to be further explored and verified in vitro.

## Conclusion

In this work, we clarified the existence of ferroptosis in the diabetic visual pathway and the role of GPX4 in controlling this process, offering a novel target for the management of diabetic visual pathway damage and the creation of neuroprotective medications.

## Supplementary Information

Below is the link to the electronic supplementary material.

Supplementary file1 (DOCX 798 KB)

## Data Availability

This is the nutstore link of all original data (including replication). https://www.jianguoyun.com/c/sd/1863c45/11cd99a43cf58ce4.
