# Supplementary figures and images for: Ferroptosis contributes to diabetes-induced visual pathway neuronal damage via iron accumulation and GPX4 inactivation
Source: Metab Brain Dis. 2024 Jul 30;39(7):1459–68. doi: 10.1007/s11011-024-01398-5 (PMC11513717; doi:10.1007/s11011-024-01398-5)

Figure 4


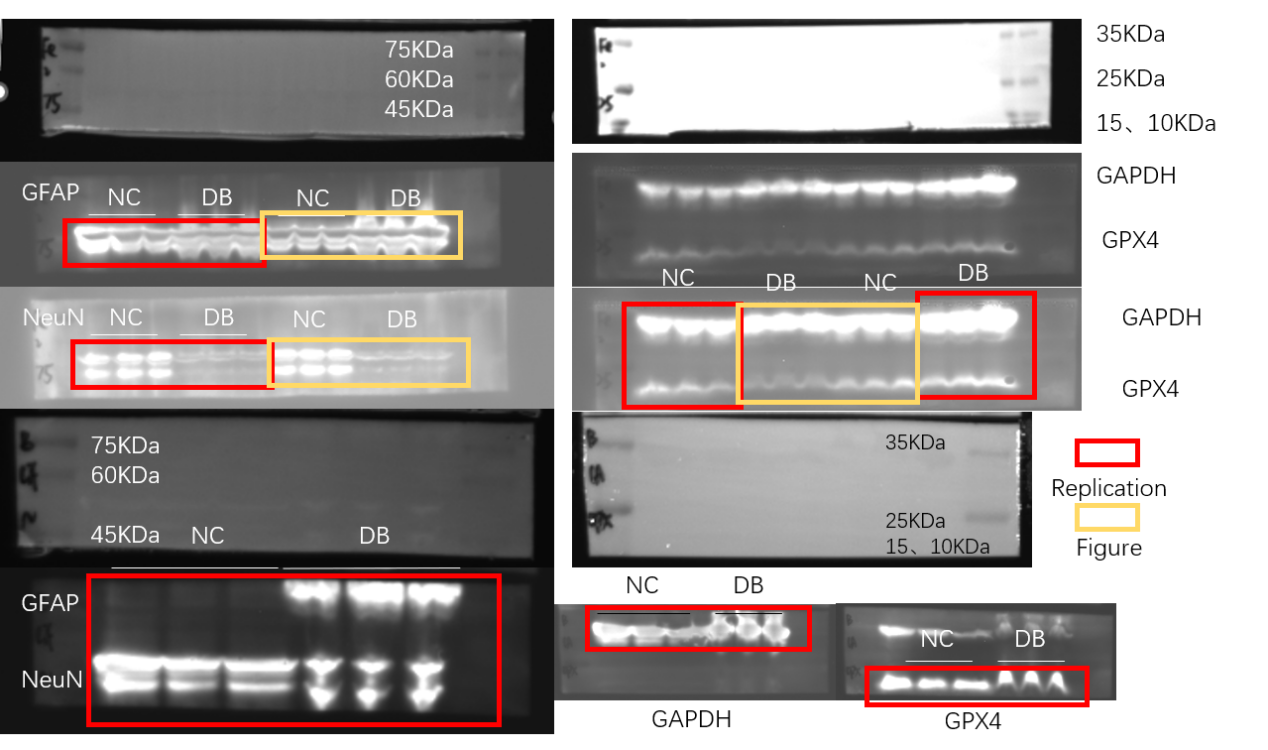


Figure 5


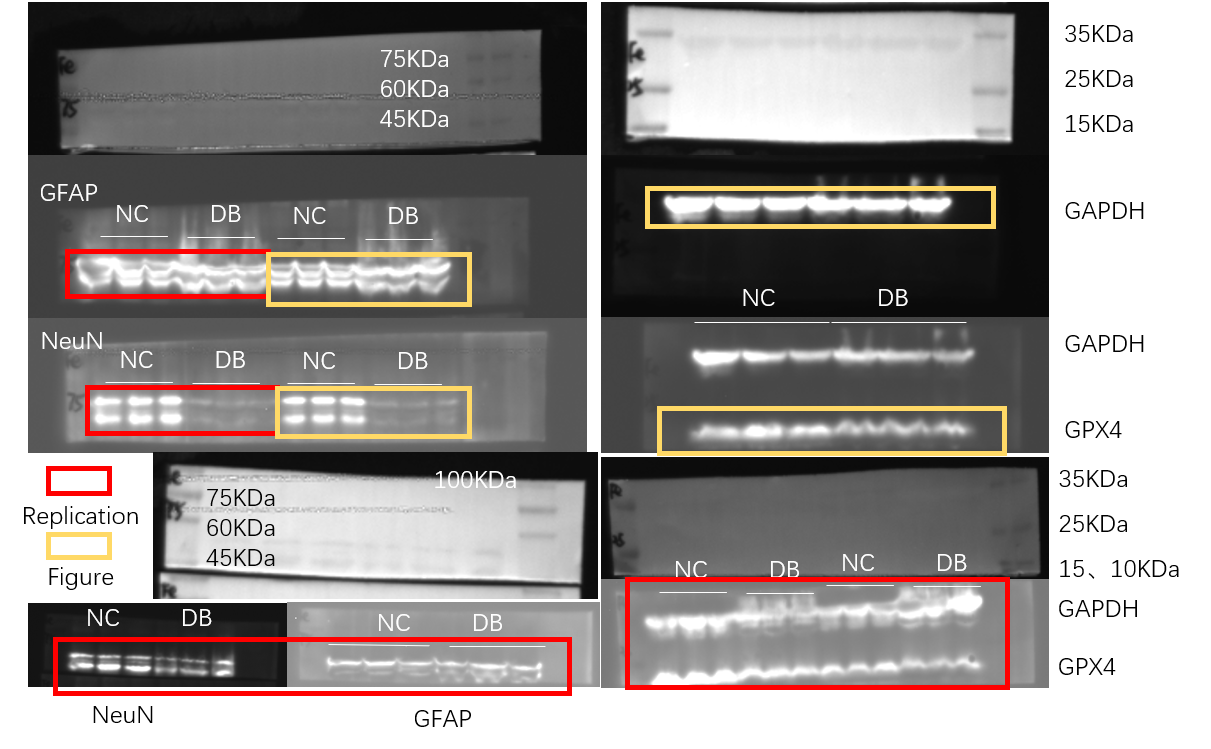

Supplement: Supplementary file 1 — Supplementary file1 (DOCX 798 KB) [file 11011_2024_1398_MOESM1_ESM.docx]
